# Biological Activity of Horehound (*Marrubium vulgare* L.) Herb Grown in Poland and Its Phytochemical Composition

**DOI:** 10.3390/ph17060780

**Published:** 2024-06-14

**Authors:** Monika Michalak, Małgorzata Stryjecka, Martyna Zagórska-Dziok, Paulina Żarnowiec

**Affiliations:** 1 Department of Pharmaceutical Sciences, Collegium Medicum, Jan Kochanowski University, IX Wieków Kielc 19, 35-317 Kielce, Poland; 2 Institute of Agricultural Sciences, State School of Higher Education in Chełm, Pocztowa 54, 22-100 Chełm, Poland; mstryjecka@panschelm.edu.pl; 3 Department of Technology of Cosmetic and Pharmaceutical Products, Medical College, University of Information Technology and Management in Rzeszów, 35-225 Rzeszów, Poland; mzagorska@wsiz.edu.pl; 4 Department of Microbiology, Faculty of Natural Sciences, Jan Kochanowski University, Uniwersytecka 7, 25-406 Kielce, Poland; paulina.zarnowiec@ujk.edu.pl

**Keywords:** *Marrubium vulgare*, essential oil, plant extract, antioxidant activity, antimicrobial properties, protective effect

## Abstract

*Marrubium vulgare* (Lamiaceae) is a plant which has long been known and used in traditional medicine for various purposes. However, few recent studies have documented its chemical composition and biological properties. The present study investigated the phytochemical composition of horehound, as well as its protective, antioxidant, and antimicrobial potential. GC-MS analysis revealed that the major components of horehound essential oil are E-caryophyllene (35.7%), germacrene D (25.2%), and bicyclogermacrene (10.6%). The biological activity of horehound hydroethanolic herb extract derives from multiple chemical compounds, including polyphenols (55.72 mg/mL), flavonoids (11.01 mg/mL), phenolic acids (4.33 mg/mL), and tannins (4.46 mg/mL). Chromatographic analyses of the extract identified 12 phenolic compounds, of which ferulic acid, catechin, quercetin, protocatechuic acid, rutin, and syringic acid (35.42, 24.69, 20.65, 18.70, 14.46, and 12.69 mg/mL, respectively) were the main constituents. Its DPPH radical scavenging ability was 68.29%, while its antioxidant properties, determined by the FRAP method, were at the level of 1.22 mmol/L. Moreover, *M. vulgare* extract decreased the level of intracellular reactive oxygen species in the fibroblasts and keratinocytes in vitro, achieving the strongest antioxidant effect at a concentration of 2.5% in the case of both types of skin cells. Extracts from the horehound herb showed significant antimicrobial and anti-biofilm activity, confirming the plant’s potential in therapeutic applications against various microbial pathogens (gram-positive and gram-negative bacteria and fungi). The research results demonstrate the protective effect of horehound extract on the viability of both fibroblasts and keratinocytes in vitro. To sum up, *M. vulgare*, as a valuable natural material with high preventive and therapeutic effectiveness, is a potential candidate for new applications in the pharmaceutical and cosmetics industries.

## 1. Introduction

The search for new plants is ongoing, as is research into plants that have long been used in order to determine their phytochemical composition and assess their biological properties and potential uses for human health. Particular attention is focused on sources of natural bioactive molecules (e.g., flavonoids, phenolic acids, tannins, and terpenoids) with significant preventive and therapeutic benefits. As a rich source of biologically active compounds, both medicinal and aromatic plants can be used as health-promoting agents in the food, pharmaceutical, and cosmetic industries [1]. The family Lamiaceae includes plants rich in bioactive substances of biological interest with important pharmacological attributes [2]. One species of this family, long known and used in traditional medicine for various purposes, is *Marrubium vulgare.* Horehound (also called white horehound or common horehound) is a plant with high bioactive potential, native to Europe, North Africa, southwestern Africa, and Central Asia [3,4]. It is a perennial plant that grows to a height of 80 cm. Its stem is sparsely branched and covered with dense grey downy hairs. The rounded and heart-shaped lower leaves and ovoid upper leaves are crinkled, with grey hairs underneath. The flowers are small, white, and bilabiate, grouped in spherical pseudowhorls in the leaf axils. The raw material is the herb (*Marrubi herba*). The flowering aerial parts are collected from June to August. The phytochemical constituents of the herb are diterpenes (labdanes), predominantly marrubiin, phenolic acid derivatives of the phenylpropanoid group, flavonoids (apigenin, luteolin, and quercetin) and their glycosides, tannins, phytosterols (e.g., β-sitosterol), triterpenes (e.g., ursolic acid), organic acids, and mineral salts [3,4,5,6]. The major components of *M. vulgare* essential oils include germacrene D, β-caryophyllene, β-bisabolene, bicyclogermacrene, and carvacrol [7]. Horehound has a musky odour, which becomes a pungent but pleasant scent when it dries. *M. vulgare* has been reported to exhibit pharmacological activities such as expectorant, tonic, diuretic, diaphoretic, antinociceptive, antispasmodic, antihypertensive, antidiabetic, gastroprotective, antihepatotoxic, anti-inflammatory, wound-healing, antiproliferative, and immunomodulatory activity. Moreover, horehound is known for its antioxidant and antimicrobial activity due to the presence of flavonoids, tannins, terpenes, and phenols [3,4,5,6].

The present study was carried out to determine the content of biologically active chemical compounds and confirm the protective, antioxidant, and antimicrobial activity of hydroethanolic extracts of the *M. vulgare* herb. 

## 2. Results and Discussion

Medicinal and aromatic plants are an important source of biologically active compounds and potential preventive and therapeutic agents. *M. vulgare* herb is a valuable and noteworthy plant material, and its chemical composition and biological properties were analysed in the present study. 

### 2.1. Chemical Composition of Essential Oil 

An analysis of *M. vulgare* essential oil revealed the presence of 31 compounds, which represent 99.9% of the total oil. The EO contained a large amount of sesquiterpene hydrocarbons (89.8%) (Table 1). 

The literature data indicate that *M. vulgare* essential oils differ in their dominant compounds depending on factors such as the country of origin of the plant and part of that country, as well as the part of the plant from which the extract was obtained [8,9,10]. The present study revealed that the major components of horehound EO were E-caryophyllene (35.7%), germacrene D (25.2%), bicyclogermacrene (10.6%), and δ-amorphene (7.2%). Other studies investigating the EO of *M. vulgare* from Poland have shown that the content of germacrene D (43.36%) was the highest in EO from the flowering plants, while E-caryophyllene (44.54%), bicyclogermacrene (20.6%), and α-humulene (5.79%) were found in higher amounts in oil from vegetative parts [11]. In EO from the flowering aerial parts of *M. vulgare* grown in Slovakia, the main constituents were β-caryophyllene (45.8%) and germacrene D (14.4%) [12]. Zarai et al. [9] analysed essential oil from the aerial parts of *M. vulgare* from Tunisia and found that the major compounds were γ-eudesmol (11.93%), β-citronellol (9.90%), citronellyl formate (9.50%), and germacrene D (9.37%). Other researchers from Tunisia reported that the main constituents of EO from horehound leaves were β-bisabolene (38.21%), germacrene D (10.51%), benzodioxole (8.6%), and β-caryophyllene (4.93%) [13]. An analysis by Rezgui et al. [10] led to the identification of 14 compounds in the EO of *M. vulgare*, also grown in Tunisia, with high content of eugenol (15.29%). *β*-caryophyllene (7.24–20.34 %), (*Z*)*-β-*farnesene (1.58–34.85 %), germacrene D (9.8–13.37 %), bicyclogermacrene (1.71–8.63 %), and *β*-bisabolene (0–16.68 %) were detected as major compounds in EO of *M. vulgare* from various locations in Turkey [8]. The results of an analysis of EO from Algeria demonstrated that its major components were 4,8,12,16-tetramethyl heptadecan-4-olid (16.97%), germacrene D-4-ol (9.61%), α- pinene (9.37%), phytol (4.87%), and dehydro-sabina ketone (4.12%) [14].

### 2.2. Phytochemical Constituents of Extract

The solvents used in the preparation of plant extracts play a major role in the extraction of a specific constituent. The nature of the solvent, in addition to factors such as geographic location and climatic conditions, determines which type of bioactive compound will be extracted [4]. A review of the literature reveals that various types of horehound extracts have been analysed, both aqueous [4] and organic, e.g., ethanol [15], methanol [13], ethyl acetate, or petroleum ether [4]. Phenolic compounds, for example, are well known to be soluble in polar solvents such as water and hydroalcoholic solutions; for this reason, mixtures of water and ethanol were used for extract preparation in this study. The presence of such compounds as flavonoids, phenolic acids, and tannins are primary indications as to whether the plant may exhibit free radical scavenging activity, as well as antibacterial and antifungal properties. Therefore, a phytochemical analysis was carried out to verify whether and in what amounts the *M. vulgare* herb extracts contained these classes of compounds.

Table 2 presents the chemical composition of the hydroethanolic extract of *M. vulgare* herb, determined by spectrophotometry. The results of the quantitative determination of the bioactive compounds showed that horehound extract was richer in polyphenols (55.72 mg gallic acid equivalent/mL) than flavonoids (11.01 mg catechin equivalent/mL). In previous research, Wojdyło et al. [16] demonstrated a total phenolic content of 3.86 mg gallic acid equivalent/100 g DW. Amri et al. [17] determined TPC and TFC using a UV method and obtained 6.02 mg gallic acid equivalent/g and 45.21 mg catechin equivalent/g, respectively, in methanol/water extract from air-dried leaves. Salay et al. [18], in their analysis of hydroethanolic extract from the aerial parts of horehound, reported total phenolics in the amount of 59.87 mg gallic acid equivalents/g of dry extract and total flavonoids at a level of 14.47 mg quercetin equivalents/g of dry extract. Mssillou et al. [2], in leaf extracts of *M. vulgare* L., found total phenolic contents at a level of 112.09 mg gallic acid equivalent/g in hydroacetonic extract, compared to 98.77 mg gallic acid equivalent/g in hydroethanolic extract. The total flavonoid content was also higher in the hydroacetonic extract (21.08 mg quercetin equivalent/g) compared to hydroethanolic extract (17.65 mg quercetin equivalent/g). Kabah et al. [19] showed that the contents of polyphenols and flavonoids in aqueous and methanolic leaf extracts were 32.71 and 60.41 mg gallic acid equivalent/g, respectively, for polyphenols and 26.02 and 33.81 mg quercetin equivalent/g for flavonoids. The research results obtained by Aouadhi et al. [20] showed that a methanol extract of the leaves was richer in polyphenols (26.8 mg gallic acid equivalent/g) than in flavonoids (0.61 mg catechin equivalent/g). An ethanolic leaf extract analysed by Mkaddem et al. [13] contained from 20.8 to 44.65 mg gallic acid equivalent/g total polyphenols and from 8.91 to 37.48 mg rutin equivalent/g total flavonoids, depending on the *M. vulgare* population. Hayat et al. [4] reported total concentrations of polyphenols between 0.27 and 86.91 μg gallic acid equivalent/mg and flavonoid concentrations from 6.08 to 33.82 μg quercetin equivalents/mg, depending on the location of the plant, the type of extract (aqueous or organic), and the polarity of the extracting solvent. Al-Zaban et al. [21] showed that methanolic extract from *M. vulgare* leaves was richer in polyphenols (36.8 mg gallic acid equivalent/g) than flavonoids (1.61 mg catechin equivalent/mL). Another study showed that methanol and acetone extracts from the aerial part had a total polyphenol content of 18.15 and 16.07 mg gallic acid equivalent/g, respectively, and total flavonoids at a level of 14.46 and 12.49 mg catechin equivalent/g [22]. 

In addition to polyphenols and flavonoids, in the present study, the content of phenolic acids was determined to be 4.33 mg caffeic acid equivalent/mL and condensed tannins were identified at a level of 4.46 mg delphinidin equivalent/mL. Similarly, extract from the aerial parts of *M. vulgare* tested by Tlili et al. [22] contained condensed tannins in the amount of 4.38 mg delphinidin equivalent/mL. Hydroethanolic leaf extract, examined by Mssillou et al. [2], was richer in tannins than hydroacetonic extract (65.51 and 46.96 mg ascorbic acid equivalent/g, respectively). In a study by Aouadhi et al. [20], the condensed tannin content in methanol extract from leaves was 0.086 mg rutin equivalent/mL. Hayat et al. [4] reported the lowest tannin levels in aqueous extracts, with a concentration of 6.94 μg catechin equivalent/mg, while for the organic extracts, the ethyl acetate extract had the highest tannin content, with a concentration of 252.68 μg catechin equivalent/mg, followed by the ethanol extract and then the petroleum ether and methanol extracts. Amessis-Ouchemoukh et al. [23] showed that methanol leaf extract contained more flavonoids and tannins than acetone extract.

Table 3 presents a qualitative and quantitative estimation of the active compounds analysed using the HPLC method. Six phenolic acids and six flavonoids were confirmed in the hydroethanolic horehound herb extract. 

In the present study, ferulic and protocatechuic acids were dominant among phenolic acids, and catechin and quercetin were dominant among flavonoids. Tlili et al. [22] found a markedly different profile, with relatively abundant compounds, which were not detected in our study: the main components of acetonic extract from the aerial parts were quinic acid and 4-*O*-caffeoylquinic acid. Rezgui et al. [10] reported that the predominant compounds in acetone and methanol extracts from the aerial parts were sinapic acid, quercetin, ferulic acid, *p*-coumaric acid, caffeic acid, apigenin, and luteolin. The major compounds revealed by Wojdyło et al. [16] in an aqueous methanol *M. vulgare* herb extract were caffeic acid, *p*-coumaric acid, and ferulic acid. Kabah et al. [19], in a study analysing *M. vulgare* leaf extract, the major phenolic compound that was identified was salicylic acid in both aqueous and methanolic extracts. A study by Gourich et al. [24] conducted in Morocco found that the dominant phenolic compounds in the aqueous extract of *M. vulgare* leaves were catechin, luteolin, apigenin, maleic acid, salicylic acid, caffeic acid, and vanillic acid. Al-Zaban et al. [21] tested a methanolic extract of air-dried leaves and showed that the major components were luteolin-7-*O*-D-glucoside, ferulic acid, and premarrubiin, in addition to terniflorin, cirsimaritin, amentoflavone, marruboside, and gallic acid, which were present in smaller quantities. 

The diversity of the structures of phenolic compounds is linked to their multi-faceted biological activity. The antioxidant activity of polyphenols results from various mechanisms of action, including elimination of reactive oxygen species through direct reaction, inhibition, or potentiation of the action of numerous enzymes, and the chelation of pro-oxidative metal ions. The antibacterial activity of polyphenols is also associated with the presence of hydroxyl groups, which have an affinity for proteins and act as inhibitors of bacterial enzymes. It may also be due to the induction of bacterial membrane disruption and the inhibition of processes such as biofilm formation and cell envelope, nucleic acid, and ATP synthesis [1,25]. 

### 2.3. Antioxidant Activity of Extract

In the present study, antioxidant activity was determined by two spectrophotometric methods, i.e., a DPPH free radical scavenging assay and a ferric reducing power assay (FRAP) and was also assessed as the ability of *M. vulgare* extract to reduce the intracellular level of reactive oxygen species in skin cells (keratinocytes and fibroblasts) in vitro. The ability to scavenge DPPH radicals was 68.29%, while the antioxidant properties determined by the FRAP method were at the level of 1.22 mmol/L. Amessis-Ouchemoukh et al. [23] reported a DPPH radical scavenging activity of *M. vulgare* leaves ranging from 51.90% to 97.15%. It is difficult to compare the results of the present study with other literature data and values recorded for the antioxidant activity of horehound due to differences in calculation units. However, Tlili et al. [22], for example, measured the antioxidant potential of methanol and acetone extracts of the aerial parts using the same two in vitro tests used in our study: DPPH (IC50 of 1.11 and 1.66 mg/mL, respectively) and FRAP (IC50 of 0.08 and 0.06 mg/mL, respectively). Salay et al. [18] noted significant antioxidant potential in the FRAP assay (64.07 mg ascorbic acid equivalents/g), while the concentration needed to neutralize of 50% (IC50) of generated DPPH radicals was 13.41 μg/mL. Hayat et al. [4] evaluated antioxidant activity using the DPPH and ABTS methods and showed that ethanol, methanol, and ethyl acetate extracts from *M. vulgare* leaves had higher percentages of inhibition than the petroleum ether extract. The inhibitory concentrations (IC50) ranged from 324.55 to 980 μg/mL for DPPH and from 107.85 to 890.74 μg/mL for ABTS. Kabah et al. [19] determined that methanolic leaf extract exhibited the highest DPPH free radical scavenging capacity, with an IC50 equal to 2.49 mg/mL, while the IC50 for the aqueous extract was 3.09 mg/mL. Similarly, antioxidant activity in the FRAP assay was higher for the methanolic extract (456.93 mg ascorbic acid equivalent/g) than for the aqueous extract (308.14 mg ascorbic acid equivalent/g) [19]. The authors of another study analysing methanolic leaf extract showed strong antioxidant power against DPPH (IC50 of 35 μg/mL) and ABTS (IC50 of 25 μg/mL) radicals [20]. Mssillou et al. [2], using the DPPH and FRAP assays, indicated that the hydroethanolic extract possessed better antioxidant activity (IC50 of 52.04 μg/mL and EC50 of 4.51 mg/mL) than the hydroacetonic extract (IC50 of 60.57 μg/mL and IC50 of 6.43 mg/mL) leaf extract of *M. vulgare*. 

**Figure 1:**
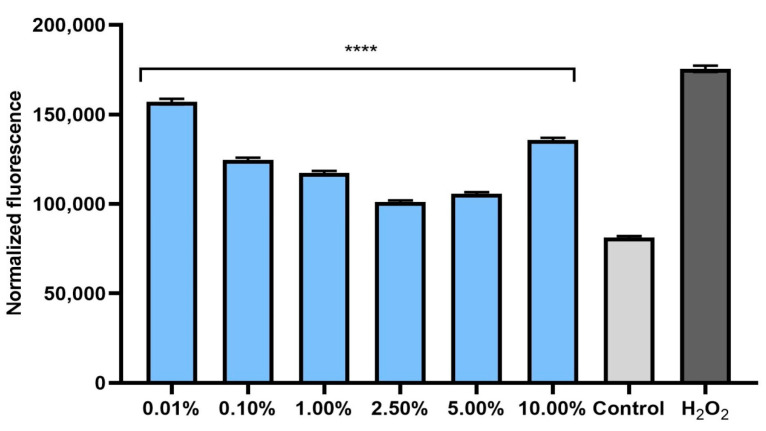
Effect of *Marrubium vulgare* herb extract (at concentrations of 0.01–10.00%) on DCF fluorescence in fibroblasts (BJ) exposed to 500 µM H_2_O_2_. Data are the mean ± SD of three independent experiments, each consisting of three replicates per treatment group. **** *p* < 0.0001 compared to positive control (cells treated with 500 µM H_2_O_2_, without the addition of the test extract).

**Figure 2:**
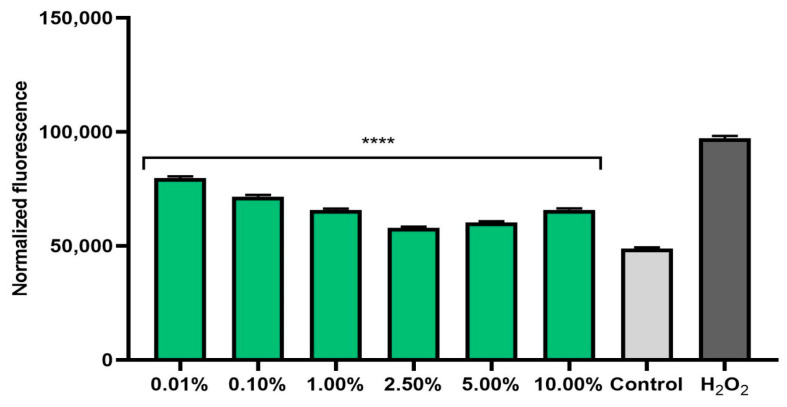
Effect of *Marrubium vulgare* herb extract (in the concentration range of 0.01–10.00%) on DCF fluorescence in keratinocytes (HaCaT) exposed to 500 µM H_2_O_2_. Data are the mean ± SD of three independent experiments, each consisting of three replicates per treatment group. **** *p* < 0.0001 compared to the positive control (cells treated with 500 µM H_2_O_2_, without the addition of test extract).

The ability of plant extracts to reduce the level of radicals in cells is extremely important in the context of protecting cells against oxidative stress. Therefore, the analyses performed in this study included a determination of the level of ROS in skin cells previously exposed to the pro-oxidant compound hydrogen peroxide (500 μM). Measurements of the iROS level were performed on human fibroblasts (BJ) and keratinocytes (HaCaT) in vitro. The effect of horehound extract on the level of iROS was shown to be strongly dependent on the concentration used. Intracellular levels of reactive oxygen species increased dramatically after hydrogen peroxide treatment for both cell types. The simultaneous exposure of cells to an extract of *Marrubium vulgare* herb caused a decrease in iROS in both fibroblasts (Figure 1) and keratinocytes (Figure 2). In both types of skin cells, as the concentration of the extract increased to 2.5%, the iROS level gradually decreased, so the strongest antioxidant effect was achieved at a concentration of 2.5%. Higher concentrations of the extract (5.0% and 10.0%) also reduced the iROS level, but as the concentration increased from 5.0%, the antioxidant effect became weaker. This trend may suggest that horehound extract at concentrations above 10% may not have a protective effect against excessive amounts of free radicals and may have a pro-oxidant effect.

The anti-radical and antioxidant properties of the water–ethanol horehound extract tested in this study is unquestionably related to the action of biologically active compounds in the herb of the plant, the presence of which was confirmed by chromatographic analysis (Table 3). These extracts contain compounds with proven antioxidant properties, such as caffeic acid, ferulic acid, catechin, quercetin, and rutin [26,27,28,29,30,31,32]. Caffeic acid is a compound that has antioxidant properties owing to its ability to prevent the formation of hydroxyl radicals, chelate metal ions, and significantly inhibit the peroxidation of membrane lipids [26,27]. Ferulic acid, due to the presence of hydroxy and phenoxy groups, effectively removes superoxide radicals and has the ability to inhibit lipid peroxidation [28,29]. Catechins are phytochemicals with the proven ability to scavenge reactive oxygen species and are chelators of metal ions. Moreover, these compounds can induce the activity of antioxidant enzymes and inhibit that of enzymes, which can increase the number of free radicals. Additionally, catechins may significantly influence the production of phase II detoxification enzymes, which may also reduce oxidative stress in cells [30]. Another powerful antioxidant present in the tested extract is quercetin, which has the ability to increase the expression level of antioxidant enzymes such as superoxide dismutase (both Cu/Zn SOD and Mn SOD), catalase (CAT), and GSH peroxidase, thereby strengthening the antioxidant defence system. Moreover, it can inhibit the activity of pro-oxidant enzymes such as acetylcholinesterase (AChE) and butyrylcholinesterase (BChE), and also reduce lipid peroxidation levels [31]. Another compound that can increase the activity of antioxidant enzymes is rutin, which can also reduce the intracellular level of free radicals [32]. Therefore, the antioxidant activity may be the result of the action of various biologically active compounds present in horehound extracts, whose activity is based on various mechanisms.

### 2.4. Antimicrobial Activity of Extract

Many plants, due to the presence of bioactive compounds, including flavonoids, tannins, and EO constituents, possess potent antimicrobial agents and provide effective remedies for skin conditions. The identification of new antimicrobials (e.g., against *Staphylococcus aureus*, a common cause of skin infections) and anti-biofilm agents is important not only for the prevention and treatment of skin diseases, but also for overall healthcare. *M. vulgare* is a plant known for its antimicrobial activity against both gram-positive and gram-negative bacteria, fungi, herpes simplex virus, and parasites, as well as its wound-healing properties [3,17,33]. In this study, *M. vulgare* extract demonstrated significant antimicrobial and anti-biofilm activity, which aligns closely with previously published data, confirming the plant’s potential in therapeutic applications against various microbial pathogens. 

The tested extracts exhibited inhibitory activity against a broad range of bacteria at concentrations ranging from 1 to 4 mg/mL. The lowest MIC value (1 mg/mL) was observed for *Candida albicans*. Our study indicates effective antibacterial activity against both gram-positive and gram-negative bacteria. MIC values of 2 mg/mL were recorded for *Enterococcus faecalis*, *Proteus mirabilis*, *Streptococcus pyogenes*, and *Streptococcus mutans*. The highest MIC values, amounting to 4 mg/mL, were noted for *Staphylococcus aureus*, *Staphylococcus epidermidis*, *Escherichia coli*, *Pseudomonas aeruginosa*, *Streptococcus agalactiae*, *Shigella sonnei*, and *Streptococcus pneumoniae* (Table 4).

This broad antibacterial spectrum is particularly significant, considering that other studies have highlighted variations based on the type of extract and the solvent used. For instance, Dib et al. [34] found that methanolic extracts of *M. vulgare* leaves had more potent antibacterial activity (against *Aggregatibacter actinomycetemcomitans* and *Eikenella corrodens*) than aqueous extracts, with MICs for methanolic extracts ranging from 1.56 to 3.12 mg/mL. Al-Zaban et al. [21] studied the antibacterial activity of the methanolic extract of horehound leaves against *Escherichia coli*, *Listeria monocytogenes*, *Staphylococcus aureus*, *Bacillus cereus*, *Enterococcus faecalis*, *Pseudomonas aeruginosa*, *Salmonella arizona*, *Salmonella typhimurium*, and *Klebsiella pneumoniae* (MIC values from 6.25 to 25 mg/mL). Masoodi et al. [35] reported that a methanolic extract of whole horehound plants exhibited moderate to significant antibacterial activity against five of the six tested bacterial species (*Escherichia coli*, *Bacillus subtilis*, *Staphylococcus aureus*, *Staphylococcus epidermidis*, *Pseudomonas aeruginosa*, and *Proteus vulgaris*). In a study by Mssillou et al. [2], a hydroethanolic extract showed activity against all tested strains, including two gram-positive bacteria (*Bacillus subtilis* and *Staphylococcus aureus*), two gram-negative bacteria (*Salmonella enterica* and *Escherichia coli*), the human fungal pathogen *Candida albicans*, and the plant fungal pathogen *Aspergillus niger.* Rezgui et al. [10] screened ethanol and acetone extracts against the dermatophyte fungi *Microsporum gypseum*, *Microsporum canis*, *Arthroderma cajetani*, *Trichophyton mentagrophytes*, *Trichophyton tonsurans*, *Epidermophyton floccosum*, and two other fungal strains (*Botrytis cinerea* and *Pythium ultimum*) [10]. Kanyonga et al. [36] reported that a whole-plant methanol extract exerted significant antibacterial activity against *Bacillus subtilis*, *Staphylococcus epidermidis*, and *Staphylococcus aureus*, was moderately effective against *P. vulgaris* and *E. coli*, but was ineffective in the case of *P. aeruginosa.* Aouadhi et al. [20] demonstrated that leaf methanol extract showed significant activity against *Bacillus cereus*, *Listeria monocytogenes*, *Staphylococcus aureus*, *E. coli*, *Pseudomonas aeruginosa*, *Aeromonas hydrophila*, and *Salmonella typhimurium*. Gourich et al. [24] described the antimicrobial activity of aqueous extract of *M. vulgare* leaves as high against *Klebsiella pneumoniae*, *Escherichia coli*, *Pseudomonas aeruginosa*, and *Staphylococcus epidermidis*, moderate against *Streptococcus agalactiae*, and low against *Proteus mirabilis*.

The anti-biofouling activity of the *M. vulgare* herb was also investigated in the present study, which revealed that the plant extract was effective across a spectrum of microbial species (Table 5). 

Biofilms are one of the major factors contributing to the progression and persistence of chronic infections, especially as the effectiveness of antibiotics becomes increasingly limited. Consequently, the search for new, effective agents to combat this issue has become a priority. Numerous studies have highlighted the inhibitory effects of flavonoids and polyphenols on bacterial biofilms [37]. The hydroethanolic extract of *M. vulgare* herb demonstrated the ability to inhibit biofilm formation over a concentration range of 4 to 16 mg/mL. Notably, at lower concentrations (4 to 8 mg/mL), the extract was effective against all the microorganisms tested except for *Staphylococcus epidermidis*, highlighting its broad-spectrum anti-biofilm activity. *Streptococcus mutans* and *Pseudomonas aeruginosa* were particularly sensitive to the extract. At concentrations of 4 to 8 mg/mL for *S. mutans* and 8 mg/mL for *P. aeruginosa*, the inhibition rate exceeded 80%. This is particularly important given the challenge posed by biofilms in clinical settings, as they significantly contribute to the persistence and resistance of infections. These findings suggest potential therapeutic applications of *M. vulgare* in the treatment and management of biofilm-associated infections.

### 2.5. Cell Viability Assay

**Figure 3:**
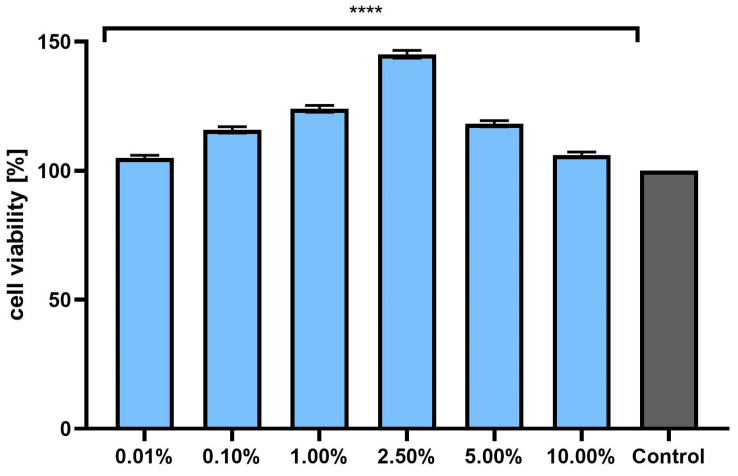
The effect of *Marrubium vulgare* herb extract (at a concentration range of 0.01–10.00%) on resazurin reduction in fibroblasts (BJ cells). The exposure time to the tested samples was 24 h. Data are the mean ± SD of three independent experiments in which each extract concentration was tested in triplicate. Control cells were cultured in DMEM medium (not exposed to the extract), for which viability was assumed to be 100%. **** *p* < 0.0001.

**Figure 4:**
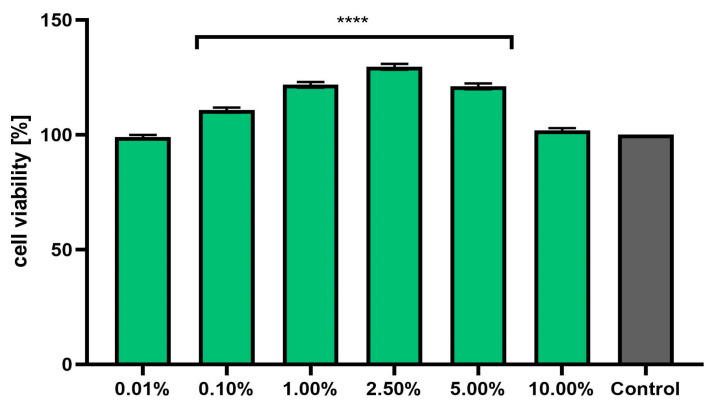
Effect of *Marrubium vulgare* herb extract (in the concentration range of 0.01–10.00%) on the reduction in resazurin in keratinocytes (HaCaT cells). The exposure time for the tested samples was 24 h. Data are the mean ± SD of three independent experiments in which each extract concentration was tested in triplicate. Control cells were cells cultured in DMEM medium (not exposed to the extract), for which the viability was assumed to be 100%. **** *p* < 0.0001.

**Figure 5:**
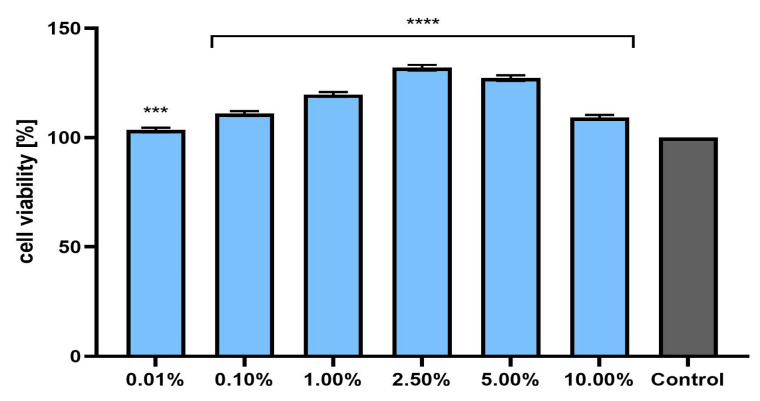
Effect of *Marrubium vulgare* herb extract (in the concentration range of 0.01–10.00%) on neutral red dye uptake in fibroblasts (BJ cells). The exposure time for the tested samples was 24 h. Data are the mean ± SD of three independent experiments in which each extract concentration was tested in triplicate. Control cells were cells cultured in DMEM medium (not exposed to the extract), for which the viability was assumed to be 100%. **** *p* < 0.0001, *** *p* = 0.0002.

**Figure 6:**
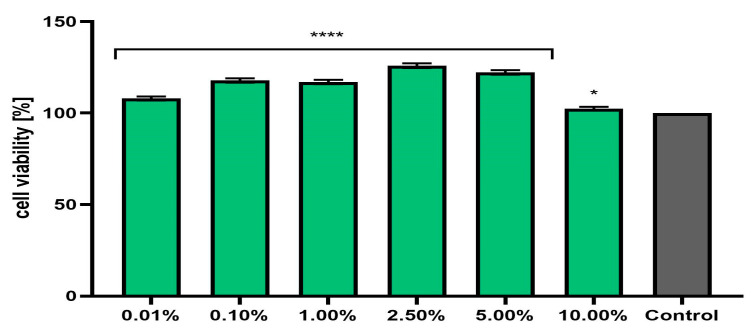
Effect of *Marrubium vulgare* herb extract (in the concentration range of 0.01–10.00%) on neutral red dye uptake in keratinocytes (HaCaT cells). The exposure time for the tested samples was 24 h. Data are the mean ± SD of three independent experiments in which each extract concentration was tested in triplicate. Control cells were cells cultured in DMEM medium (not exposed to the extract), for which the viability was assumed to be 100%. **** *p* < 0.0001, * *p* = 0.0129.

Assessment of the possibility of using various chemical compounds or plant extracts in cosmetic or pharmaceutical preparations intended for the care or treatment of skin diseases should include an assessment of their cytotoxicity to skin cells. Therefore, the study assessed the effect of horehound herb extract on the metabolic activity, proliferation, and viability of fibroblasts and keratinocytes in vitro. Two cytotoxicity assays were used for this purpose—alamar blue and neutral red (Figure 3, Figure 4, Figure 5 and Figure 6). The first test determines the reduction of the oxidized blue dye resazurin to the pink fluorescent product resorufin, which was carried out only by living cells. The second is based on measurements of the cells’ ability to take up neutral red and accumulate this dye in the lysosomes. The analyses performed in this study indicated that the extract, at the tested concentration range, showed no cytotoxicity for either type of skin cell in vitro. The effect of *M. vulgaris* herb extract on cell viability in both tests was dependent on the cell type and the concentration used. Measurements of resazurin reduction by cells exposed to the extract indicated that, in the case of fibroblasts, the most beneficial effect was obtained at a concentration of 2.5%, at which point viability increased to 145.1%. In the case of keratinocytes, the highest increase in viability (129.6%) was recorded for the extract concentration of 1.0%. In the neutral red assay, the highest viability for both cell types was observed after using a concentration of 2.5%. This concentration increased the viability of fibroblasts to 131.9% and that of keratinocytes to 125.9%. The noticeable downward trend in cell viability in both tests when higher concentrations of the extract were used (5.0% and 10.0%) may indicate that, at higher concentrations (above 10.0%), it may reduce the metabolic activity and proliferation of skin cells in vitro.

Only a few literature reports have been published regarding the effects of horehound extracts on skin cells. Amri et al. [17] used the MTT test to demonstrate that *Marrubium vulgare* extract can have a favourable effect on the proliferation of fibroblasts in vitro. The authors also indicated a protective effect of these extracts against the cytotoxic effect of DMSO on fibroblasts. Moreover, their research showed the possibility of influencing the migration of these cells and improving the wound healing process. The present study is the first to demonstrate the protective effect of extracts from this plant on the viability of both fibroblasts and keratinocytes in vitro. The positive effect on the metabolic activity and proliferation of these cells is probably linked to the activity of the biologically active compounds contained in the herb of the plant, including polyphenols. The literature reports indicate that plant polyphenols can have positive, multifaceted effects on skin cells [38,39]. The antioxidant effect of the extract tested in this work and its ability to reduce the intracellular level of reactive oxygen species undoubtedly affect the viability of the cells and protect them against the negative effects of cytotoxic compounds such as the above-mentioned DMSO and H_2_O_2_. This protective effect may suggest that these extracts could be used in products applied to the skin. However, it is important to select a concentration of *M. vulgare* extracts that would prevent the developed cosmetics or pharmaceuticals from exerting cytotoxic effects on the cells of individual skin layers.

## 3. Materials and Methods

### 3.1. Chemicals

The reagents used were deionized water, methanol, ethanol, glycerol, butanol (POCH, Gliwice, Poland), Folin–Ciocalteu (F-C) reagent, sodium carbonate, sodium nitrite, sodium hydroxide, aluminium chloride (Chempur, Piekary Śląskie, Poland), sodium acetate, ferric chloride (Warchem, Zakręt, Poland), gallic acid, 2,4,6-tripyridyl-s-triazine (TPTZ), 1,1-diphenyl-2-picrylhydrazyl (DPPH), hydrochloric acid, Arnova reagent, caffeic acid, delphinidin, catechin, *Trolox* (Sigma Aldrich, Saint Louis, MO, USA), brain–heart infusion broth (BHI, Graso, Owidz, Poland), and the antibiotics streptomycin, erythromycin, and fluconazole (Sigma-Aldrich, Poznań, Poland). The standards for HPLC analysis were caffeic acid, ferulic acid, ellagic acid, protocatechuic acid, *p*-coumaric acid, syringic acid, catechin, quercetin, myricetin, apigenin, luteolin, and rutin (Sigma-Aldrich, Saint Louis, MO, USA). Cell culture and in vitro tests were performed using the following sterile reagents: DMEM medium with 4500 mg/L glucose, L-glutamine and sodium pyruvate (Dulbecco’s Modification of Eagle’s Medium, Biological Industries, Genos, Łódź, Poland), antibiotics (penicillin-streptomycin, Life Technologies, Bleiswijk, The Netherlands), ethanol (C_2_H_5_OH, 96%, Sigma-Aldrich, Poznań, Poland), acetic acid (CH_3_COOH, Pol-Aura, Morąg, Poland), Fetal Bovine Serum (FBS, Biological Industries, Genos, Łódź, Poland), phosphate-buffered saline solution (PBS, pH 7.00 ± 0.05, Chempur, Piekary Śląskie, Poland), neutral red dye solution (NR, 0.33%, Sigma-Aldrich, Poznań, Poland), resazurin sodium (RES, Sigma-Aldrich, Poznań, Poland), and trypsin-EDTA solution with phenol red (Sigma-Aldrich, Poznań, Poland). 

### 3.2. Plant Material 

The aerial parts of horehound (*Marrubium vulgare*) were harvested in June 2023, during the flowering stage, from the Botanical Garden in Kielce (51°7′ N, 23°28′ E, Poland). A voucher specimen was deposited at the Herbarium KPC, Jan Kochanowski University, Kielce, Poland. The plant material was dried in a convection oven (Binder FD 53, Tuttlingen, Germany) in an air stream at 35 °C and ground in a mill (A11 basic, IKA-Werke, Staufen, Germany). 

### 3.3. Essential Oil Separation and GC-MS Analysis

Essential oil was extracted from the dried herb via water distillation for three hours using a Clevenger apparatus. All essential oil samples were stored in a refrigerator at 4 °C until an analysis of their composition was conducted using gas chromatography (Agilent Technologies 7890B, Santa Clara, CA, USA) in conjunction with mass spectrometry (Agilent Technologies 5977A, Santa Clara, CA, USA). The HP Innowax Agilent 19091N-116 capillary column (60 m × 0.320 mm and 0.25 μm) was used. The carrier gas was helium, with a flow rate of 1.3 mL/min. To analyse the composition of the essential oils, they were dissolved in hexane (20 μL of essential oil was diluted in 1 mL of n-hexane). The analysis was performed in split mode (40:1). The injection volume was 1 μL, and the injection temperature was set to 250 °C. The initial temperature of the column after injection was 70 °C, which was held for 5 min and then increased to 160 °C (3 °C/min), and this temperature was held for 5 min. Finally, the temperature was increased to 250 °C, at 6 °C/min, and held for 5 min. The detector and ion source temperatures were 270 °C and 230 °C, respectively. Retention indices were determined by injecting C7–C30 n-alkanes (Sigma-Aldrich, St. Louis, MO, USA) into the system (GC/FID) (Agilent Technologies, 7890B, Santa Clara, CA, USA) in the same conditions as for GC-MS analysis. Constituents were identified by comparing the spectra from the MS detector with spectra from the Wiley and NIST libraries and with the available literature data [40,41,42]. The percentage content of constituents was determined by GC-FID analysis.

### 3.4. Extract Preparation

The extracting solvent (50/50 EtOH/water solution (*v*/*v*), 60 mL) was added to 2 g of powdered plant material. Extraction was performed twice for 60 min using an ultrasonic bath (Polsonic 5, Warsaw, Poland). The resulting extracts were filtered with Whatman filter paper.

### 3.5. Total Polyphenol Content of Extract

The total polyphenol (TP) content was estimated by the Folin–Ciocalteu method [43]. Standard solutions of gallic acid with a precisely known concentration were used to construct a calibration graph. The analysis was performed in triplicate. The results were expressed as mg of gallic acid equivalents (GAE) per mL of each extract. The absorbance was determined spectrophotometrically at 765 nm using the UV-1900i UV–Vis spectrophotometer (Shimadzu, Kyoto, Japan).

### 3.6. Total Flavonoid Content of Extract

The total flavonoid (TF) content was estimated according to Kim et al. [44]. Standard solutions of catechin with a precisely known concentration were used to construct a calibration graph. The analysis was performed in triplicate. The results were expressed as mg of catechin equivalents (CE) per mL of extract. The absorbance was determined spectrophotometrically at 510 nm using the UV-1900i UV–Vis spectrophotometer (Shimadzu, Kyoto, Japan). 

### 3.7. Total Phenolic Acid Content of Extract

The total phenolic acid (TPA) content was assessed according to Jain et al. [45]. Standard solutions of caffeic acid with a precisely known concentration were used to construct a calibration graph. The analysis was performed in triplicate. The results were expressed as mg of caffeic acid equivalents (CAE) per mL of each extract. The absorbance was determined spectrophotometrically at 490 nm using the UV-1900i UV–Vis spectrophotometer (Shimadzu, Kyoto, Japan).

### 3.8. Condensed Tannin Content of Extract

The condensed tannin (CT) content was estimated according to Tlili et al. [22]. Standard solutions of delphinidin with a precisely known concentration were used to construct a calibration graph. The analysis was carried out in triplicate. The results were expressed as mg of delphinidin equivalents (DpE) per mL of each extract. The absorbance was determined at 550 nm spectrophotometrically using the UV-1900i UV–Vis spectrophotometer (Shimadzu, Kyoto, Japan).

### 3.9. HPLC Analysis

An HPLC analysis of flavonoids and phenolic acids was carried out with the Advanced Prominence-i (Shimadzu, Kyoto, Japan) and a SunFire C18 (150 mm × 4.6 mm, 5 μm; Waters, Milford, CT, USA) analytical column at ambient temperature. The mobile phase consisted of water with 0.1% formic acid (A) and acetonitrile (B), and the flow rate was 1 mL/min. The injection volume was 10 μL, and the compounds were detected with a UV multi-wave detector. Compounds were identified by comparing the retention times and calibration curves with those of standard substances. The validation parameters are presented in Supplementary Table S1. Each sample was analysed in triplicate. The results were presented as arithmetic mean ± standard deviation (SD).

### 3.10. DPPH Scavenging 

The anti-radical power of the extract was measured by the widely used DPPH test [46]. Briefly, 50 μL of extract or ethanol (as a blank) was mixed with 0.5 mL DPPH solution and left to stand for 20 min in the dark. The following formula was used to calculate the degree of inhibition of DPPH radical by the sample: DPPH scavenging activity (%) = [A_0_ − A_1_/A_0_] × 100, where A_0_ is the absorbance of the control and A_1_ is the absorbance of the sample. All experiments were carried out in triplicate, and the results were reported as means ± SD of triplicates. Free radical scavenging activity is expressed as the percentage decrease in DPPH. The absorbance was determined at 517 nm using the UV-1900i UV–Vis spectrophotometer (Shimadzu, Kyoto, Japan).

### 3.11. Ferric Reducing Antioxidant Power (FRAP Assay)

The reducing power of the extracts was determined according to the method described by Benzie and Strain [47]. Briefly, the FRAP reagent (prepared by mixing acetate buffer (300 mM, pH 3.6), a solution of 10 mM TPTZ in 40 mM HCl, and 20 mM FeCl_3_ at 10:1:1 (*v*/*v*/*v*)) was mixed with a specified concentration of the plant extract and incubated at 37 °C for 4 min. A standard curve was prepared using different concentrations of Trolox. The results were expressed as mmol of Trolox equivalent (TE) per L of each extract. Each sample was prepared in triplicate. The absorbance was determined at 593 nm using a UV-1900i UV–Vis spectrophotometer (Shimadzu, Kyoto, Japan).

### 3.12. Cell Culture

Tests of cytotoxicity and the ability of the *M. vulgare* herb extract to reduce the level of intracellular reactive oxygen species (iROS) were performed on two skin cell lines. These cells were BJ human fibroblasts (American Type Culture Collection, Manassas, VA 20108, MA, USA) and HaCaT keratinocytes (CLS Cell Lines Service GmbH, Eppelheim, Germany). Both cell lines were cultured in Dulbecco’s Modified Eagle Medium (DMEM, Biological Industries, Cromwell, CO, USA). DMEM medium with a high glucose content (4.5 g/L) was additionally supplemented with sodium pyruvate and the amino acid L-glutamine. To provide the cells with the appropriate amounts of nutrients, 10% foetal bovine serum (Merck Life Science, Darmstadt, Germany) was added during the culturing. To prevent the multiplication of microorganisms in the lines, antibiotics (100 U/mL penicillin and 1000 µg/mL streptomycin) were added in the amount of 1% (Gibco, Waltham, MA, USA). The culturing was carried out in an incubator at 37 °C in a humid atmosphere containing 95% air and 5% carbon dioxide. Cells were passaged with trypsin–EDTA solution after reaching approximately 70–80% confluence. All reagents used during culture were sterile.

#### 3.12.1. Cytotoxicity Analysis—Alamar Blue (AB) and Neutral Red (NR) Uptake Assays

The resazurin reduction test (AB) and the neutral red uptake assay (NR) were used to assess the cytotoxicity of the horehound extract on skin cells (BJ fibroblasts and HaCaT keratinocytes). The analyses were performed as previously described [48]. Briefly, cells seeded in 96-well plates were incubated for 24 h with *M. vulgare* herb extract at concentrations of 0.01%, 0.1%, 1.0%, 2.5%, 5.0%, and 10.0%. Individual concentrations were prepared by dissolving the extract in complete DMEM culture medium. Cells untreated with extracts, to which only culture medium was added, were used as a control.

In the case of the AB assay, the individual extract solutions were removed from the wells after 24 h of incubation. Next, resazurin solution (60 μM) was added to each well. Then, after 2 h incubation at 37 °C, the fluorescence in each well was quantified at λ = 570 nm using a FilterMax F5 microplate reader from Thermo Fisher Scientific (Waltham, MA, USA). For the NR test, after removing the individual extract dilutions from the wells, neutral red dye (40 µg/mL) was added. The cells were then incubated with the dye for 2 h at 37 °C. After this time, the neutral red dye was removed and the cells were washed twice with sterile PBS. To release the dye from the lysosomes, 150 μL of destaining buffer (C_2_H_5_OH/CH_3_COOH/H_2_O, 50%/1%/49%) was then added. The absorbance of the samples was measured at a wavelength of λ = 540 nm using the above-mentioned microplate reader. Three independent experiments were performed, in which each dilution of the extract was tested in triplicate.

#### 3.12.2. Determination of Intracellular Level of Reactive Oxygen Species (ROS)

The ability of the *M. vulgare* herb extract to reduce oxidative stress in keratinocytes and fibroblasts exposed to 500 µM H_2_O_2_ was assessed by measuring the intracellular level of reactive oxygen species in skin cells. The analyses were performed according to a previously described method [48]. First, skin cells (keratinocytes and fibroblasts) were seeded in 96-well plates. The cell density in each well was 1 × 10^4^ cells. After the cells had attached to the bottom of the plates, they were treated with 500 µM H_2_O_2_ and horehound extract in a concentration range of 0.01–10.00% for 24 h. Cells treated with H_2_O_2_ alone were used as a positive control, and cells exposed to neither hydrogen peroxide nor to the extract were used as a negative control. After 24 h of incubation, 180 µL of H_2_DCFDA solution (10 µM) was added to the wells with the cells. After 60 min of incubation with the fluorescent probe, fluorescence measurements were made in each well using a Filter Max UV–Vis spectrophotometer reader (Thermo Fisher Scientific, Waltham, MA, USA). Measurements were made at an excitation wavelength of λ = 485 nm and an emission wavelength of λ = 530 nm. Three independent experiments were performed for each type of skin cells. Each sample was tested in triplicate in each experiment.

### 3.13. Evaluation of the Antimicrobial Activity of the Extracts

#### 3.13.1. Test Microorganisms

The antimicrobial properties of the extracts were evaluated against 12 microorganisms, listed in Table 6, sourced from the Polish Collection of Microorganisms, Wroclaw, Poland.

#### 3.13.2. Minimum Inhibitory Concentration (MIC)

A quantitative assay of the antibacterial activity of the extracts was performed using the broth microdilution method, according to the guidelines of the Clinical and Laboratory Standards Institute, in order to establish the MICs. An overnight culture of bacteria and fungi was diluted at a ratio of 1:10 with fresh BHI broth. A 100 μL volume of the extracts at a concentration of 33 mg/mL was added to the first well of a 96-well microtiter plate and serially diluted 1:1 with inoculated broth at final concentrations of 0.25 to 16 mg/mL. Then, the plates were incubated at 37 °C for 24 h, and the absorbance was measured at 600 nm using an Infinite M200 PRO microplate reader (Tecan, Männedorf, Switzerland). The positive controls were streptomycin for bacteria and fluconazole for fungi, at concentrations ranging from 0.1 to 250 µg/mL. If the bacteria were not susceptible to streptomycin, erythromycin was used instead. All experiments were conducted in triplicate. The MIC values were determined as the lowest concentration of extract at which no microbial growth was observed [49].

#### 3.13.3. Minimum Bactericidal/Fungicidal Concentration (MBC/MFC)

The MBC/MFC was determined by plating a 100 µL aliquot on solid culture BHI medium from the last three wells, which showed inhibited microbial growth in the MIC assay. The plates were incubated at 37 °C for 24 h. The MBC was defined as the lowest concentration at which a 99.9% reduction in living microbial cells was recorded. All experiments were run in triplicate.

#### 3.13.4. Inhibition of Biofilm Formation

Biofilm formation was quantified by the method described by Merrit et al. [50], in 100 µL of BHI in 96-well, flat-bottom microplates for 24 h at 37 °C, in the presence of extract at final concentrations from 0.25 to 16 mg/mL. Following incubation, the media with suspended bacteria were removed, the wells were washed with water, and 0.1% crystal violet solution was added for 15 min. After removing the dye solution and washing with water, the attached dye was solubilized with 95% ethanol and brief vortexing. The amount of biomass was measured at 595 nm using an Infinite M200 PRO microplate reader (Tecan, Männedorf, Switzerland). A 10% bleach solution was used as a positive control. Each treatment was carried out in triplicate. The total amount of cells attached in the form of biofilm to the wells of the microtiter plate was directly proportional to the amount of dye extracted from the wells. The percentage of biofilm inhibition was calculated using the following formula [51]:

% biofilm inhibition = (control OD value − treated OD value/control OD value) × 100

### 3.14. Statistical Analysis

The experiments were replicated three times, and the data were expressed as the average ± standard deviation (SD). In the case of tests on cell lines, a statistical analysis of the results was performed using GraphPad Prism 8.4.3 software (GraphPad Software, Inc., San Diego, CA, USA). For this purpose, a two-way analysis of variance (ANOVA) was used, and post hoc intergroup Dunnett’s test was performed. Statistical significance was determined for significance levels of **** *p* < 0.0001, *** *p* < 0.001, ** *p* < 0.01, and * *p* < 0.05 compared to the control.

## 4. Conclusions

The results of the research revealed that horehound may be used as a valuable source of natural bioactive molecules with high preventive and therapeutic effectiveness, especially in skin care and treatment. The *M. vulgare* hydroethanolic extract was shown to have potent protective properties for skin cells and to exhibit antioxidant and antimicrobial activity. Further research is required to evaluate the practical benefits of preventive and therapeutic applications of horehound extract.

## Figures and Tables

**Table 1:** Chemical composition of *Marrubium vulgare* essential oil.

Compound	RT ^a^	RI ^b^	% ^c^
α-Thujene	7.948	857	0.1 ± 0.00
β-Pinene	7.993	862	0.1± 0.00
Limonene	8.712	942	1.1 ± 0.03
γ-terpinene	9.772	1060	1.2 ± 0.03
Geijerene	10.508	1142	0.4 ± 0.02
*trans*-Pinocamphone	10.670	1160	0.1 ± 0.00
*cis*-Pinocamphone	10.796	1174	0.1± 0.00
Thymol	11.002	1197	0.5 ± 0.02
Carvacrol	11.065	1204	5.7 ± 0.06
α-Copaene	11.811	1287	1.9 ± 0.02
β-Bourbonene	11.910	1298	2.1 ± 0.03
β-Elemene	12.098	1319	1.3 ± 0.03
E-Caryophyllene	12.197	1330	35.7 ± 0.05
β-Copaene	12.260	1341	2.8 ± 0.04
β-Gurjunene	12.305	1342	0.1 ± 0.00
(Z)-β-Farnesene	12.529	1367	0.1 ± 0.00
α-Humulene	12.610	1376	0.1 ± 0.00
Germacrene D	12.691	1385	25.2 ± 0.06
Viridiflorol	12.754	1392	0.1 ± 0.00
Bicyclogermacrene	12.826	1400	10.6 ± 0.04
γ-Cadinene	12.988	1418	0.1 ± 0.00
δ-Amorphene	13.131	1434	7.2 ± 0.02
α-Cadinene	13.293	1452	0.1 ± 0.00
β-Sesquiphellandrene	13.832	1512	0.1 ± 0.00
α-Cadinene	14.066	1538	0.1 ± 0.00
E-Nerolidol	14.398	1575	0.1 ± 0.00
Spathulenol	14.497	1586	0.1 ± 0.00
Caryophyllene oxide	14.533	1590	0.1 ± 0.00
Humulene epoxide II	14.784	1618	0.1 ± 0.00
epi-α-Muurolol	14.910	1632	0.1 ± 0.00
α-Cadinol	15.054	1648	0.5 ± 0.00
Total			99.9 ± 0.15
Monoterpene hydrocarbons			2.9 ± 0.04
Oxygenated monoterpenes			6.4 ± 0.07
Sesquiterpene hydrocarbons			89.8 ± 0.12
Oxygenated sesquiterpenes			0.8 ± 0.05

^a^ RT, retention time (minute); ^b^ RI, the Kovats retention index; ^c^ percentage composition of a compound.

**Table 2:** Phytochemical composition of *Marrubium vulgare* extract.

Total Polyphenols (mg GAE/mL ± SD)	Total Flavonoids (mg CE/mL ± SD)	Total Phenolic Acids (mg CAE/mL ± SD)	Condensed Tannins (mg DpE/mL ± SD)
55.72 ± 0.06	11.01 ± 0.03	4.33 ± 0.03	4.46 ± 0.05

GAE, gallic acid equivalent; CE, catechin equivalent; CAE, caffeic acid equivalent; DpE, delphinidin equivalent; SD, standard deviation.

**Table 3:** HPLC analysis of *Marrubium vulgare* herb extract.

	Compound	Content (mg/mL ± SD)
Phenolic acids	Caffeic acid	0.67 ± 0.04
	Ferulic acid	35.42 ± 0.07
	Protocatechuic acid	18.70 ± 0.02
	*p*-Coumaric acid	16.65 ± 0.04
	Ellagic acid	0.15 ± 0.00
	Syringic acid	12.69 ± 0.01
Flavonoids	Catechin	24.69 ± 0.02
	Quercetin	20.65 ± 0.03
	Myricetin	0.79 ± 0.01
	Apigenin	2.31 ± 0.01
	Luteolin	6.68 ± 0.02
	Rutin	14.46 ± 0.01

**Table 4:** MIC and MBC/MFC values of *Marrubium vulgare* extract and standard drugs in mg/mL.

Test Microorganism	Hydroethanolic Extract		Streptomycin/ Erythromycin	Fluconazole
	MIC	MBC/MFC	MIC	MIC
*Staphylococcus aureus*	4.0 ± 0.02	4.0	0.001 ± 0.005	-
*Staphylococcus epidermidis*	4.0 ± 0.04	4.0	0.062 ± 0.04	-
*Streptococcus agalactiae*	4.0 ± 0.01	4.0	0.001 ± 0.02	-
*Streptococcus mutans*	2.0 ± 0.03	2.0	0.004 ± 0.03	-
*Streptococcus pyogenes*	2.0 ± 0.12	2.0	0.001 ± 0.02	-
*Streptococcus pneumoniae*	4.0 ± 0.05	4.0	0.001 ± 0.02	-
*Enterococcus faecalis*	2.0 ± 0.002	2.0	0.004 ± 0.02	-
*Escherichia coli*	4.0 ± 0.05	4.0	0.015 ± 0.04	-
*Pseudomonas aeruginosa*	4.0 ± 0.08	4.0	0.015 ± 0.02	-
*Shigella* *sonnei*	4.0 ± 0.01	4.0	0.015 ± 0.02	-
*Proteus mirabilis*	2.0 ± 0.09	2.0	0.062 ± 0.07	-
*Candida albicans*	1.0 ± 0.14	1.0	-	0.001 ± 0.02

MIC, Minimum Inhibitory Concentration; MBC, Minimum Bactericidal Minimum Fungicidal Concentration; MFC, Minimum Fungicidal Concentration. The positive controls were streptomycin for bacteria and fluconazole for fungi. If the bacteria were not susceptible to streptomycin, erythromycin was used instead, - not tested.

**Table 5:** Percent of biofilm-forming inhibition of *Marrubium vulgare* extract against various microorganisms.

Strain	Extract Concentration (mg/mL)						
	0.25	0.5	1	2	4	8	16
Gram-positive bacteria							
*S. aureus*	17.7 ± 2.8	14.1 ± 0.9	30.8 ± 3.7	34.9 ± 1.6	49.2 ± 1.3	38.2 ± 2.0	-
*S. epidermidis*	4.4 ± 0.3	-	-	-	-	-	-
*S. agalactiae*	-	-	-	-	58.3 ± 0.9	51.8 ± 2.3	-
*S. mutans*	-	-	-	-	83.67 ± 0.6	80.57 ± 2.6	60.7 ± 2.1
*S. pyogenes*	-	-	17.1 ± 3.7	60.6 ± 3.2	72.5 ± 0.8	68.9 ± 2.1	27.8 ± 4.1
*S. pneumoniae*	-	-	-	-	64.9 ± 1.0	59.5 ± 2.6	-
*E. faecalis*	-	-	27.5 ± 4.0	42.7 ± 0.3	42.1 ± 1.0	42.9 ± 0.3	-
Gram-negative bacteria							
*E. coli*	21.4 ± 0.7	32.0 ± 0.2	38.29 ± 1.2	31.5 ± 1.0	42.6 ± 1.2	58.7 ± 1.1	-
*P. aeruginosa*	-	-	-	47.5 ± 2.2	66.0 ± 5.0	81.3 ± 1.7	57.9 ± 2.7
*S.* *sonnei*	27.5 ± 0.7	18.5 ± 1.7	29.3 ± 1.3	26.4 ± 0.3	13.6 ± 1.8	1.4 ± 4.0	-
*P. mirabilis*	-	19.3 ± 2.4	48.2 ± 1.2	38.2 ± 1.7	60.4 ± 5.1	65.2 ± 2.8	31.1 ± 2.0
Fungal strain							
*C. albicans*	-	53.0 ± 0.6	57.1 ± 2.4	67.3 ± 0.7	65.5 ± 0.8	64.0 ± 1.8	23.7 ± 1.8

-, no inhibition. The positive control was a 10% bleach solution, which resulted in 100% inhibition.

**Table 6:** Pathogenic bacteria and fungi used for the antimicrobial assay.

Bacterial Strains	
Gram-Positive	Gram-Negative
*Staphylococcus aureus* PCM 2054	*Pseudomonas aeruginosa* PAO1
*Staphylococcus epidermidis* PCM 2118	*Escherichia coli* PCM 2057
*Streptococcus agalactiae* PCM 2683	*Proteus mirabilis* S1959
*Streptococcus pyogenes* PCM 2855	*Shigella sonnei* PCM 2336
*Streptococcus mutans* PCM 2502	
*Streptococcus pneumoniae* PCM 2589	
*Enterococcus faecalis* PCM 2673	
Fungal strains	
*Candida albicans* ATTC 10231	

## Data Availability

The data presented in this study are available on request from the corresponding author.

## Supplementary Materials

The following supporting information can be downloaded at: https://www.mdpi.com/article/10.3390/ph17060780/s1, Table S1. Calibration curves, limit of detection, and limit of quantification for standards.
