# Supplementary material for: Biological Activity of Horehound (Marrubium vulgare L.) Herb Grown in Poland and Its Phytochemical Composition
Source: Pharmaceuticals (Basel). 2024 Jun 14;17(6):780. doi: 10.3390/ph17060780 (PMC11206634; doi:10.3390/ph17060780)
Supplement: Supplementary file 1 [file pharmaceuticals-17-00780-s001.zip › pharmaceuticals-3030000-supplementary.pdf]

Supplementary Table S1:

Table S1. Calibration curves, limit of detection, and limit of quantification for standards

Compound	Calibration curve	R^2	LOD	LOQ
Caffeic acid	Y=5.0224x-0.1607	0.9974	0.9885	2.9957
Ferulic acid	Y=5.1333x+0.205	0.9929	1.6280	4.9335
Protocatechuic acid	Y=5.0811x+0.7314	0.9947	1.4109	4.2754
p-Coumaric acid	Y=5.1524x-0.7407	0.9928	1.6370	4.9607
Ellagic acid	Y=2.2823x+0.7386	0.9917	1.7615	5.3380
Syringic acid	Y=5.1532x-1.6982	0.9940	1.4962	4.5341
Catechin	Y=6.3212x+1.7546	0.9979	0.8868	2.6874
Quercetin	Y=6.2699x+5.0668	0.9952	1.3355	4.0470
Myricetin	Y=4.2361x-2.0736	0.9870	2.2087	6.6932
Apigenin	Y=4.3073x-2.7489	0.9742	3.1337	9.4961
Luteolin	Y=3.9957x+2.5068	0.9992	0.5329	1.6150
Rutin	Y=2.5682x+2.6843	0.9670	3.5568	10.7783

LOD, limit of detection; LOQ, limit of quantification
